# Extraction Techniques for Brewer’s Spent Grain Protein: A Comparative Review of Efficiency, Purity, and Functionality

**DOI:** 10.3390/foods14234058

**Published:** 2025-11-26

**Authors:** Haocheng Tong, Puxuan Zhang, Liang Zhang, Wei Zhou, Zhengte Lin, Tengfei Yu, Guanchen Liu, Donghong Liu

**Affiliations:** 1 College of Biosystems Engineering and Food Science, Zhejiang University, Hangzhou 310058, China; 2 Innovation Center of Yangtze River Delta, Zhejiang University, Jiaxing 314100, China; 3 Technology Research Institute, CR Snow Breweries, Beijing 100010, China

**Keywords:** alternative protein sources, BSGP, industrial by-product valorization, functional properties, sustainable extraction technologies

## Abstract

Brewer’s spent grain (BSG), the primary by-product of beer production, represents a promising and sustainable source of plant-based protein. This review provides a comprehensive overview of extraction techniques for brewer’s spent grain protein (BSGP), encompassing conventional methods—such as alkaline, hydrothermal, ethanol, and enzymatic extraction—as well as emerging green approaches, including ultrasound-assisted, microwave-assisted, subcritical water, and deep eutectic solvent extraction. The influence of key extraction parameters on protein yield, purity, and structural integrity is critically examined, along with the resultant alterations in functional properties such as solubility, emulsifying capacity, foaming ability, and gelation behavior. Although through parameter optimization and the application of novel technology, the existing research has been able to increase the protein extraction rate and achieve better functional properties, the challenges of obtaining higher protein purity and extracting proteins on a larger scale remain. Collectively, these findings underscore the considerable potential of BSGP as a multifunctional ingredient in next-generation sustainable food formulations.

## 1. Introduction

**Figure 1:**
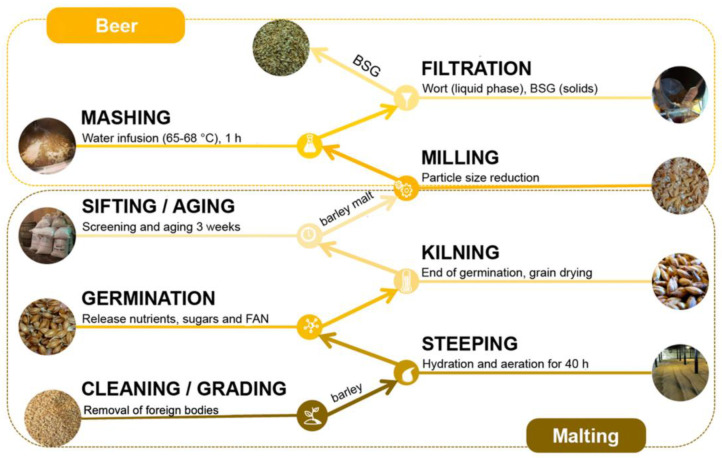
BSG production of barley grain [7].

The growing global population and the finite nature of resources have imposed significant pressure on industries to adopt sustainable development practices. There is an ongoing global effort to minimize processing waste and increase up-cycling of processing side-streams in order to support sustainable growth in the coming decades [1]. In the food industry, beer, as one of the most popular beverages globally, has a significant and continuously expanding market size [2]. The beer production process generates substantial amounts of by-products, among which brewer’s spent grain (BSG) is the most prominent. It is the solid residue obtained from barley after mashing and filtration from the brewing process [3]. Once the wort is used for fermentation, BSG no longer participates in subsequent production processes [4]. This process is shown in Figure 1. For every 100 L of beer produced, approximately 20 kg of BSG is generated, accounting for 85% of all by-products from the brewing industry [5]. Currently, BSG is mainly used in animal feed, composting, and biogas production [6]. However, the growing demand for the high-value utilization of by-products has prompted people to explore more efficient ways for the utilization of BSG.

BSG primarily consists of barley husks, bran, remnants of the endosperm, and other grains used in beer production [8]. It is rich in lignin, cellulose, hemicellulose, lipids, and proteins [4], while also containing small amounts of starch and ash. Its detailed composition is outlined in Table 1. Due to differences in the BSG sources, the malt types, and the analytical methods applied, the compositional proportions reported in different studies may vary. However, they generally fall within a stable range.

Among the various components of BSG, brewer’s spent grain protein (BSGP) has garnered significant attention. With the increasing world population, global demand for protein will continue to increase. Current statistics indicate that protein sourced from animals constitutes 40% of protein consumed worldwide, and it will increase to 73% in 2050 [16]. Conventional meat production will no longer suffice to cater to the increasing demand for food from the increasing population. This dilemma has urged researchers to find sustainable protein resources from non-traditional origins [17]. Plant-based proteins not only align with consumers’ growing preference for natural, animal-free, and health-conscious products [18] but may also be sourced from industrial by-products, offering environmentally friendly alternatives for human diets [15]. BSG, being a by-product which features high production levels as well as protein content between 15% and 30% on a dry matter basis, stands out as an extremely viable source of plant protein [4]. It offers a sustainable, as well as high-quality protein resource that fulfills human requirements for health [19]. Research has identified that BSGP constitutes largely hordein, glutelin, globulin, as well as albumin [10], with the hordein being its most dominant fraction at 43%, followed by glutelin at 21.5% [20]. In addition, the amino acid composition of BSGP further reflects its nutritional potential. Rich in both essential and non-essential amino acids, BSGP shows a high content of glutamic acid (about 12–24%) and proline (6–14%) [10,17,21], which are the major amino acids in hordein and glutelin. Relatively high levels of branched-chain amino acids such as leucine and valine were also reported, while lysine remains the limiting amino acid [10,17,21], as is typical for cereal proteins [22]. Overall, this amino acid profile highlights the potential of BSGP for food applications.

While BSG has been applied to increase protein content in staple foods, for example, in bread and pasta, its antinutritional factors may suppress nutrient absorption [23]. For this reason, extracting BSGP might provide more versatile life applications in the food industry, as well as a more improved nutritional value. However, its rigid structural composition, cell walls, as well as its vacuoles impart major setbacks for effective protein extraction [9].

Various extraction techniques have been explored to overcome these challenges. However, a comprehensive review that directly links extraction methods with extraction yield, protein purity, and the techno-functional properties of BSGP is still lacking at present. To address this gap, this review systematically examines extraction methods for BSGP, compares in detail their extraction yields and protein purities, and explains how key processing parameters influence protein functional properties. This study aims to provide a solid foundation for future research and practical applications, highlighting the potential of BSGP as a sustainable protein source.

## 2. Extraction Methods for BSGP

As the insoluble residue obtained after wort filtration, BSG contains a high moisture content of approximately 75–80%. To preserve its quality and extend its shelf life, it is essential to remove moisture through a drying process. The moisture content of dried BSG should not exceed 10% [24]. Drying not only prevents microbial spoilage but also significantly reduces the volume of BSG, thereby facilitating storage and transportation [8]. Researchers usually oven-dried BSG at temperatures between 45 °C and 60 °C [21,25,26], and air drying was also used in some studies [11].

**Figure 2:**
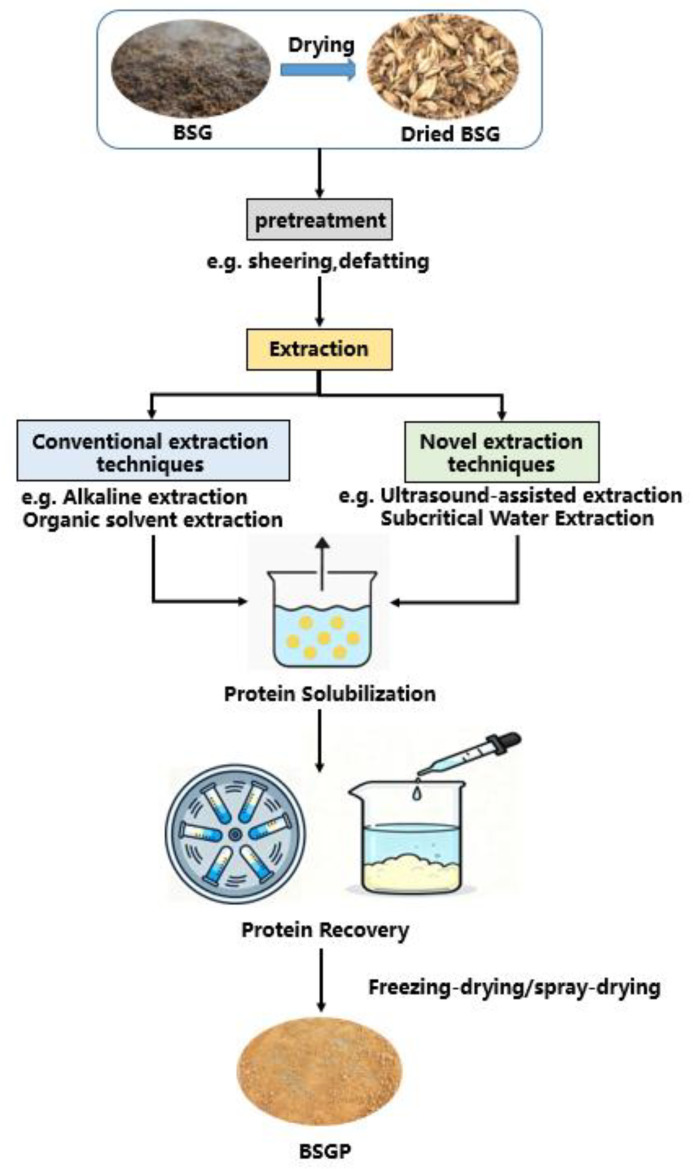
Schematic of the overall extraction process for brewers’ spent grain protein.

Following the drying process, a variety of pretreatment methods have been employed, often aimed at removing impurities and facilitating the release of proteins from the BSG matrix [4]. Subsequently, different protein extraction technologies have been designed as well as utilized, as tabulated in Table 2, including extraction methodology, processing conditions, as well as resultant outputs. While different factors, such as BSG source and assay methods, can influence the results, the overall composition of BSG in most studies is similar, allowing for comparisons across research [27]. Generally, the extraction of BSGP usually consists of two main stages: protein solubilization as well as protein recovery. The BSG is first mixed with water-based solutions, which may contain a variety of reagents such as water, acid, alkali, and organic solvents. These reagents help disintegrate the BSG matrix and decrease interactions between proteins and other macromolecules, thus facilitating their release. [4]. After removing the solid residues by centrifugation, the recovered proteins are then isolated using isoelectric precipitation, followed by centrifugation or ultrafiltration. Then, Solid protein particles were finally obtained by freeze-drying or spray-drying. The overall process for BSGP extraction is presented in Figure 2.

This section reviews different extraction methods and the variations in their processing parameters. The effectiveness of these methods is compared using two key indicators:

Extraction Yield, defined as the proportion of protein recovered relative to the total protein content in BSG.

Protein Purity, defined as the proportion of protein in the isolated fraction.

### 2.1. Pretreatment

Pretreatment enhances extraction efficiency and contributes to improving the yields of both conventional and novel techniques [2]. Despite numerous studies on the effects of pretreatment on extraction performance, their conclusions often vary.

Shearing as a pretreatment has proven beneficial, likely due to the physical disruption of cell membranes and lignocellulosic material, which facilitates protein extraction [1]. Connolly et al. [10] observed that shearing speed and duration significantly influence protein yield. For gray BSG, shearing at 11,000 rpm for 20 or 60 s tripled the protein yield. Similarly, for black BSG, shearing at 11,000 rpm for 20 or 60 s increased the extraction rate by 1.4 times. Although Qin et al. [14] suggested that shearing prior to alkaline treatment had no significant impact on protein extraction efficiency, numerous studies have adopted shearing as a pretreatment, presumably because physical comminution facilitates disruption of the BSG matrix and promotes protein release into solution.

Also, several studies use ultrasound-assisted pretreatment to physically break down the BSG matrix, which utilizes the cavitation effect caused by high-frequency sound waves to disrupt cell wall structures through mechanical vibration, accelerating solvent penetration and protein diffusion. Junttila [25] demonstrated that ultrasonic pretreatment significantly improved protein extraction from BSG.

To disrupt the BSG matrix, dilute sulfuric acid pretreatment and carbohydrases hydrolysis have also been employed. Dilute acid pretreatment can hydrolyze cellulose and hemicellulose, facilitating the separation of proteins from other polysaccharides. Junttila [25] and Qin et al. [14] reported that dilute sulfuric acid pretreatment significantly increased protein extraction efficiency. 

As a pretreatment, carbohydrases hydrolysis can disrupt the polysaccharide matrix of BSG. Niemie et al. [11] addicted that the application of Depol 740 L (a carbohydrate cell-wall–degrading enzyme cocktail, notably with feruloyl esterase and xylanase activities) markedly improved the release of protein into solution. However, Zhang et al. [30] reported that cellulase pretreatment resulted in a lower protein extraction yield compared to direct alkali extraction (66.41%→50.48%).

As a critical part of the plant protein extraction process [40], defatting is also a common pretreatment method in BSGP extraction, because BSG contains a certain amount of lipids (5–14%). It helps remove lipids and improve the extraction purity of proteins. Various solvents have been used to defat brewers’ spent grain. Qin et al. [14] used n-hexane for defatting, while Karlsen et al. [29] utilized a methanol-chloroform mixture, and Wahlström et al. [39] employed supercritical carbon dioxide. Despite the adoption of defatting pretreatment in several studies, Karlsen et al. argued that defatting may reduce protein extraction efficiency and has no significant effect on purity, as water-soluble proteins may dissolve in the solvent or undergo structural changes during the defatting process.

Delignin presents significant challenges in the extraction of BSG protein due to the strong interactions between lignin and proteins, which are difficult to separate. Rommie et al. [41] suggested that covalent interactions might exist between lignin and proteins, as the addition of antioxidants prior to acid precipitation did not reduce lignin-protein co-precipitation, indicating that disulfide bonds are not involved. Similarly, Niemi et al. [11] reported the co-precipitation of lignin and proteins. Karlsen et al. [29] used a 60% ethanol aqueous solution (1:4, *w*/*v*), heated under reflux at 180 °C for 90 min for delignification. While this treatment had little effect on total protein extraction yield, it significantly improved protein purity by reducing the co-extraction of lignin and lignin-hemicellulose complexes. However, this method may result in the loss of some alcohol-soluble proteins.

Fungal fermentation pretreatment enhances the protein content in BSG and improves the purity of the extracted protein products. Chin et al. [31] applied Rhizopus oligosporus for fermentation pretreatment, increasing the protein content of BSG from 25.2% to 34.1%. Additionally, the purity of proteins extracted using an ethanol-sodium sulfite solution improved from 60.7% to 66.2%. Chai and Chen [17] also utilized this fungus for fermentation pretreatment. Previous studies have used fungal fermentation to treat BSG, increasing its protein content and promoting BSG proliferation [42,43]. Applying this method as a pretreatment for BSG protein extraction may enhance extraction efficiency, as the degradation of polysaccharides and improved solubility, caused by partial hydrolysis of proteins, contribute to the enhanced extraction.

### 2.2. Conventional Extraction Technologies

Traditional extraction methods have long been the cornerstone of protein extraction from BSG. These methods are widely used due to their simplicity, cost-effectiveness. Even though they may not always reach the same level of efficiency as more advanced techniques, these approaches are still preferred in many industrial applications due to their proven effectiveness, scalability, and availability of equipment [15,29].

Below, we will discuss the three most commonly used traditional extraction methods for BSG protein extraction: Alkaline Extraction, Ethanol Extraction, and Hydrothermal Extraction.

#### 2.2.1. Alkaline Extraction

Alkali extraction is currently the most widely used and well-known method for protein extraction [1]. This approach employs alkaline solutions, such as NaOH, under varying conditions of concentration, solid-to-liquid ratio (SSR), temperature, and treatment time. It is highly efficient, even for insoluble proteins, as the alkaline environment increases the surface charge of hydrophobic proteins, enhancing their solubility in water. Additionally, alkaline conditions disrupt plant cell structures, facilitating protein solubilization and release. So this method is cost-effective and relatively simple [12,44,45].

It has been widely applied to extract BSGP. Key variables determining extraction efficiency include the type and concentration of alkali, extraction temperature, SSR, extraction time, and the isoelectric point for protein precipitation.

NaOH is the most commonly used alkali, and its concentration significantly affects extraction. For example, Junttila et al. [25] reported that 0.05 M NaOH yielded lower extraction rates than 0.1 M solutions. Similarly, Silva et al. [15] found that raising pH levels increased extraction rates significantly, with pH 11 identified as optimal (an extraction yield of 87%). A higher extraction pH and temperature result in more severe disruption of the brewer’s spent grain matrix, which is beneficial for the extraction of BSGP. These results suggested that alkaline conditions could enhance the protein extraction, probably by disrupting hydrogen bonding, solubilizing protein–cell wall complexes, and facilitating the dissociation of proteins from lignocellulosic matrices [4].

SSR has an inverse relationship with extraction yield; a lower SSR increases yield but often reduces purity due to the dissolution of more impurities. Temperature also plays a crucial role: higher temperatures accelerate mass transfer and reduce cell wall resistance, increasing protein solubility but potentially reducing purity and causing protein denaturation. Junttila et al. [25] observed that as the extraction temperature increased from 40 °C to 80 °C, protein yield improved, but protein purity decreased. Similarly, Karlsen et al. [29] reported a similar trend at 30 °C, 45 °C, and 60 °C over 30 min of extraction. These results indicated that higher temperature promoted protein solubilization by increasing cell wall permeability and molecular mobility [15]. However, temperatures higher than 60 °C may cause protein denaturation, making them unsuitable for protein extraction [46]. Extraction time also influences extraction yield, as Junttila et al. [25] pointed out that longer extraction time could raise the yield.

After protein solubilization through alkaline extraction, acid precipitation is commonly used to recover the dissolved proteins [47]. This method relies on the isoelectric point (pI) of proteins, which represents the pH at which a protein carries no net charge. At pH values above the pI, proteins carry a net negative charge, leading to electrostatic repulsion, while below the pI, they carry a net positive charge, resulting in similar repulsive forces. At the pI, the balance of positive and negative charges minimizes repulsion and promotes intermolecular attraction, causing protein aggregation and precipitation [48]. Typically, researchers use a pH range of 2 to 4.5 for protein precipitation. This is due to the presence of different proteins found in BSG that are known to have isoelectric points ranging from pH 6 (hordeins) to around pH 3.7–4.5 (glutelins) [4].

#### 2.2.2. Hydrothermal Extraction and Ethanol Extraction

Hydrothermal extraction uses water as a solvent under mild to high temperatures for varying durations to facilitate protein release [49]. In this process, heated water helps dissolve proteins by partially breaking down the complex cell wall structures found in biomass. However, since the BSG matrix is quite resistant to breaking down and BSGP has low water solubility, the overall extraction efficiency of this method remains limited. Du et al. [28] reported an extraction yield of only 6.8%, indicating that more strong processing conditions are required for effective extraction of BSGP.

Ethanol extraction has a high application for extracting bioactive compounds [50], including BSGP. It exploits ethanol’s selective solvent characteristic for alcohol-soluble proteins, which results in a good separation of it from other elements like polysaccharides. Due to high hordein content in BSG, it is relatively efficient for protein extraction. It, however, has a low capability for break down the BSG matrix, as well as low efficiency in protein extraction from gluten [12], resulting in limited extraction efficiency.

#### 2.2.3. Enzymatic Extraction

Enzymes are protein molecules that are capable of catalyzing biological reactions [51]. Enzymatic extraction utilizes carbohydrate-degrading enzymes or proteases to degrade carbohydrate and protein, forming protein hydrolysates and enhancing protein solubility [12,32]. Carbohydrases are found to be efficient in cell wall disruption of plants with minimal extensive physical or chemical treatments [52]. Proteases like Alcalase will strongly warrant protein extraction enhancement by degrading structural proteins intertwined with cell wall polysaccharides, thereby facilitating the release of proteins from the cellular matrix [53,54]. Compared with conventional alkali extraction, enzymatic extraction operates under lower temperatures and milder pH conditions. In addition, with minimal protein damage, it facilitates retention of more of the bioactive as well as functional properties [11]. Under moderate hydrolysis conditions, protease treatment can improve protein solubility and generate more bioactive peptides [55], thereby enhancing its value. Enzymatic protein extraction yield of BSGP varies between 31% as well as 86%, with typical purity between 40% as well as 45%.

In enzymatic extraction, carbohydrases are commonly used to pretreat BSG. It breaks down the cell wall structure and enhances the subsequent release of proteins. Studies have shown that pretreatment with cellulases, such as Depol 740, followed by the addition of proteases like Alcalase, significantly improves protein solubility and extraction efficiency [11,32]. Selecting appropriate enzymes and combinations, as well as optimizing pH, temperature, enzyme concentration, and reaction time, are critical to achieving optimal extraction performance. For example, Kriisa et al. found that using both Protamex and Flavourzyme resulted in better protein solubility and functionality [13]. Temperature and pH also have an important effect on enzymatic extraction. Enzymes require optimal pH and temperature conditions to exhibit their full catalytic activity [56]. Additionally, optimizing the solid-to-liquid ratio and enzyme concentration is essential. A higher liquid-to-solid ratio may dilute enzyme concentration and slow down the reaction, while a low ratio could increase impurities and lower protein purity [33].

### 2.3. Novel Extraction Technologies

**Figure 3:**
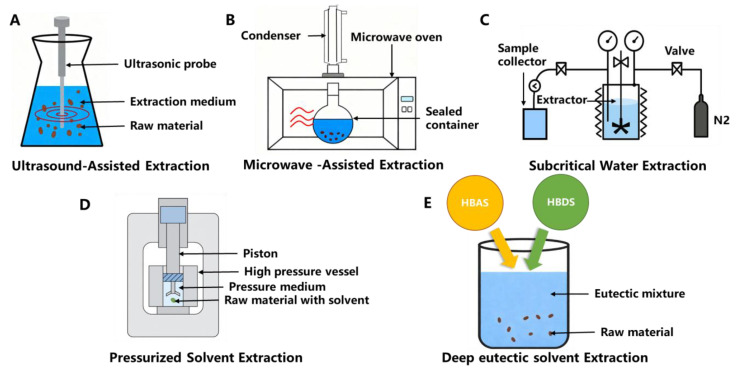
Novel extraction technologies. Ultrasound-assisted extraction (**A**), Microwave-assisted extraction (**B**), Subcritical water extraction (**C**), Pressurized solvent extraction (**D**), Deep eutectic solvent extraction (**E**).

In recent years, innovative extraction methods have been developed to improve the yield, purity, and functionality of the proteins extracted from BSG. These methods typically employ advanced physical or chemical techniques to enhance protein solubilization and separation from the BSG matrix. Figure 3 shows the schematic of the novel extraction method.

#### 2.3.1. Ultrasound-Assisted Extraction

Ultrasound-assisted extraction (UAE) is a non-thermal technique often combined with alkali extraction or enzymatic hydrolysis to improve protein yields and functional properties [57,58]. Cavitation effects, including bubble implosions, microjets, and particle collisions, enhance extraction efficiency [59]. For example, Li et al. [26] reported a protein yield increase from 46% to 86% after 20 min of ultrasound at 250 W during alkali extraction. Increased ultrasonic power and time further improve yields by intensifying cavitation and enhancing cell wall permeability [60]. However, excessive sonication may cause protein aggregation and co-precipitation of residual impurities [61]. So moderate ultrasonic duration and power will be sufficient to achieve the preferable extraction efficiency.

#### 2.3.2. Microwave-Assisted Extraction

Microwave-assisted extraction (MAE) is an economical, efficient, and straightforward method for protein extraction [37]. Microwaves, operating at frequencies between 300 MHz and 300 GHz, induce molecular vibrations through electromagnetic energy, facilitating molecular separation [62]. As an environmentally friendly technique, MAE uses little energy, works quickly, and reduces the use of harmful chemicals. Microwaves heat polar molecules inside the material, creating local heat and pressure at the cell wall. This process breaks the cells and helps release proteins [62]. It has been applied to extract proteins from peanut flour, mustard, and green coffee beans [62,63,64]. For BSGP, Barrios et al. [62] achieved a 93.99% extraction yield using MAE combined with alkali extraction. Similarly, Chai and Chen [17] reported an 82.2% yield by combining MAE with three-phase partitioning for fungal-fermented BSG, which was much higher than that from three-phase partitioning alone (41.8%).

#### 2.3.3. Subcritical Water Extraction

Subcritical water extraction uses water as the solvent but involves pressurized systems to keep it in liquid form at temperatures above 100 °C [21,65]. Under these conditions, water exhibits unique properties. It has a higher ionic product and a lower dielectric constant, which makes it act more like a low-polarity organic solvent. This helps the extraction happen faster and increases the yield [66]. Existing studies suggest that subcritical water extraction has potential as a method capable of achieving high yields. For example, researchers achieved extraction yields of 64% and 78% using subcritical water extraction, and validated the process at a pilot scale [21,38].

#### 2.3.4. Pressurized Solvent Extraction

Pressurized solvent extraction utilizes solvents under high temperature and pressure conditions [34]. These conditions enhance solvent penetration into the sample matrix. Consequently, compared to traditional solid-liquid extraction methods, pressurized solvent extraction requires significantly less solvent. Additionally, it improves extraction yield and reduces processing time. While pressurized solvent extraction is primarily applied for the extraction of small molecules, such as phenolic compounds, it can also be employed for protein extraction. Previous studies have successfully used pressurized solvent extraction for extracting proteins from pomegranate and algae [67,68]. For instance, González-García et al. [34] achieved a 69% extraction yield using ethanol as the solvent.

#### 2.3.5. Deep Eutectic Solvent Extraction

Deep eutectic solvents (DESs), as the new kind of green solvents, were first proposed by Abbott [69]. DESs are low-melting mixtures consisting of hydrogen bond acceptors (HBA) and hydrogen bond donors (HBD) with a certain stoichiometric ratio. They are composed of safe, cheap, renewable, and biodegradable compounds, which have the advantage of being more green and environmentally friendly [70]. Wahlström et al. [39] demonstrated the use of DES as an effective alternative for protein extraction. Their system consisted of 90 wt.% sodium acetate (NaAcO) and urea in a molar ratio of 1:2, supplemented with 10 wt.% water. BSG solids were mixed with the solvent at a solid-to-solvent ratio of 1:9 (*w*/*w*) and stirred at 80 °C for 2 h, yielding a protein extraction rate of 79% and a purity of 52–54.7%. This research shows that DES systems can achieve high extraction efficiency while keeping good protein purity.

## 3. Property Modification by Extraction Techniques

Apart from nutritional value and bioactivity, the functional properties of proteins are also crucial in food applications. In this review, we use the term “techno-functional properties” to denote attributes that stem from the intrinsic physicochemical characteristics of proteins [71,72]. In this section, we analyze how different extraction methods influence the structural features of BSGP and how these structural changes influence its techno-functional performance, providing a basis for its practical application.

### 3.1. Structural Properties Modification by Extraction Techniques

#### 3.1.1. Molecular Weight Distribution

The extraction conditions significantly influence the protein composition. Connolly et al. [10] found in BSG extracts at 50 °C, 58.8% of the proteins had a molecular weight greater than 10 kDa. At 20 °C, 72% of the proteins in the pale BSG extracts exhibited a molecular weight greater than 10 kDa. This conclusion indicates that higher extraction temperatures result in proteins with lower molecular weights. Additionally, other studies have indicated that ultrasound-assisted extraction methods result in proteins with smaller molecular weights, while microwave-assisted extraction did not change the molecular weight [17,26]. Additionally, enzymatic treatment results in a smaller molecular weight distribution, as it generates some small peptides [11,12]. Although research on other plant proteins suggests that higher pH values lead to smaller molecular weights [72], there is evidence in the literature that the extraction pH has no significant effect on the molecular weight distribution of brewer’s spent grain protein [73]. Overall, stronger extraction conditions generally result in smaller molecular weights, as the generation of more small peptides [26].

#### 3.1.2. Secondary Structure

Studies have shown that as the extraction pH increases, the β-turn content of BSG proteins decreases, while the content of random coil increases, indicating that the elevated extraction pH promotes protein unfolding [73]. The protein unfolding could be attributed to the higher net negative charges possessed by protein molecules when they were extracted at higher alkaline pH. The larger net charges led to increased repulsive forces between the deprotonated amino acid chains in the protein, which in turn triggered protein unfolding [74].

The secondary structures of alkaline-extracted BSGP and ethanol-extracted BSGP show clear differences related to the extraction method [12]. BSGP obtained through alkaline extraction has a more open structure with more intramolecular β-sheets. This finding suggests that alkaline extraction promotes protein unfolding. On the other hand, ethanol-extracted BSGP tends to form intermolecular aggregates, resulting in less defined secondary structures. These structural differences indicate that the alkaline extraction method enhances protein exposure and flexibility, while ethanol extraction leads to more aggregated and less soluble protein structures.

Li et al. [26] noted that ultrasound treatment led to an increase in the β-sheet content, along with a decrease in the content of α-helix, β-turn, and random coil. The α-helix structure is the most tightly connected structure within protein molecules; therefore, the reduction in α-helix content indicates the stretching of BSGP molecules [75]. As a result, under the current ultrasound treatment conditions, the protein structure becomes looser, exhibiting softness and flexibility.

#### 3.1.3. Surface Hydrophobicity

Surface hydrophobicity characterizes the exposed hydrophobic residues in a protein and is a function of the protein structure and amino acid composition. Silva et al. [15] pointed out that surface hydrophobicity (H0) of proteins was significantly influenced by extraction pH, with higher pH values increasing hydrophobicity due to protein unfolding and exposure of hydrophobic residues. Elevated temperatures also contributed to increased surface hydrophobicity by partially denaturing the proteins. This conclusion is similar to a previous research of quinuo protein [76].

Extraction methods further influenced hydrophobicity based on protein composition. Alkaline extraction, which solubilizes both hordeins and glutelins, led to the highest hydrophobicity because it contains more hydrophobic amino acids. Ethanol extraction, primarily solubilizing hordeins, resulted in lower hydrophobicity due to protein aggregation. Enzymatic extraction, which produces smaller peptides, showed the lowest hydrophobicity, as the smaller peptides exposed fewer hydrophobic groups [12]. Thus, the extraction method and protein composition play key roles in determining surface hydrophobicity for specific applications.

### 3.2. Techno-Functional Properties Modification by Extraction Techniques

The influence of extraction conditions on the techno-functional properties of BSGP is summarized in Table 3 and will be examined in detail in the following sections.

#### 3.2.1. Protein Solubility

Protein solubility is critical for the functionality of any protein because it affects other properties such as emulsifying, foaming, and gelation. Solubility reflects how many protein molecules remain dissolved in a given medium and is an excellent indicator of a protein’s state in specific environments [78,79].

Several studies have investigated the solubility of BSGP. Its solubility depends strongly on pH. In general, BSGP has very low solubility in the acidic to neutral pH range, which is common in food systems [4,10]. The solubility reaches its minimum at the isoelectric point (pI), and increases as the pH rises. For example, Chai and Chen [17] reported that BSGP solubility was below 10% at pH 4, but exceeded 80% at pH 10.

Extraction conditions also have a strong effect on solubility. Studies have indicated that the solubility is influenced by the extraction pH [15]. Mild extraction pH can yield proteins with better solubility without disrupting their native conformation. Corresponding to the previous analysis, higher extraction pH values increase the surface hydrophobicity of proteins. These proteins aggregate due to intermolecular hydrophobic interactions and disulfide bonds, leading to a decrease in their solubility [80]. In addition, temperature impacts the solubility of BSGP. Generally, increasing temperature enhances protein solubility by promoting molecular motion and interactions with the solvent. Silva et al. [15] found that BSGP solubility increased as the temperature rose within a suitable range. Under mild extraction conditions (pH 8, 60 °C), the solubility of BSGP reached about 83% when tested at pH 7 and room temperature.

The amino acid composition and molecular weight of BSGP also influence its solubility. Proteins rich in polar or charged amino acids are generally more soluble in aqueous phases. Kriisa et al. and Yu et al. [13,58] demonstrated that enzymatic hydrolysis significantly improved protein solubility, with solubility exceeding 90% after enzymatic treatment.

In addition to enzymatic hydrolysis, certain extraction methods have also been shown to enhance BSGP solubility. Techniques such as microwave processing [17], fungal fermentation [31], and ultrasound-assisted extraction [26] have all contributed to improved solubility of BSGP.

#### 3.2.2. Water-Holding and Oil-Holding Capacities

Water-holding capacity (WHC) refers to a protein’s ability to retain water within its three-dimensional structure, while oil-holding capacity (OHC) describes a protein’s ability to absorb, retain, and interact with lipids. These properties play a crucial role in oil distribution and emulsification, significantly impacting food systems [79]. Both WHC and OHC depend on the polarity and non-polarity of protein side chains, as well as the conformation and surface hydrophobicity of proteins or their aggregates [12,81].

Studies indicate that extraction methods and processing conditions significantly affect the WHC and OHC of BSGP. Extraction pH and temperature are factors in determining WHC, while they have no significant effect on oil-holding capacity. Silva et al. [15] found that increasing the extraction pH from 8 to 12 significantly improved the WHC of BSGP, from 3.2 g/g to 5 g/g, due to compositional changes in the protein extracts. Moreover, extraction temperatures ranging from 40 °C to 80 °C did not significantly affect the WHC (*p* < 0.05), suggesting that pH plays a more critical role in enhancing water retention.

Chin et al. [31] observed that BSGP hydrolysates from solid-state fermentation demonstrated significantly improved WHC (4.0 g/g), higher than non-fermented BSGP. Their OHC reached approximately 7.2 g/g, twice that of non-fermented BSGP. This improvement is attributed to the increased number of polar groups, such as COOH and NH2, in hydrolysis products during fermentation, which enhance water adsorption [82].

Chin et al. [12] further compared BSGP extracted via alkali and alcohol methods, obtaining WHC values of 4 g/g and 2 g/g, respectively. As a partially unfolded protein, alkali-extracted BSGP contains exposed polar amino acid side chains, enabling it to retain more water than alcohol-extracted proteins. Alkali-extracted proteins also contain high-molecular-weight glutenins, which form a network capable of retaining more water compared to the alcohol-extracted proteins. The OHC values for alkali and alcohol-extracted BSGP were 2.3 g/g and 2.9 g/g, respectively.

Innovative extraction techniques like ultrasound-assisted extraction and microwave-assisted extraction also significantly affect the WHC and OHC. Li et al. [26] reported that UAE of BSG proteins resulted in a WHC of 4.5 g/g, similar to proteins extracted by traditional methods. However, the ultrasound treatment enhanced the OHC to 3.1 g/g, due to the exposure of hydrophobic groups buried within the protein molecules. This reduction in α-helix content, which contributes to protein unfolding, allows for better adsorption at water-oil interfaces. Similarly, microwave-assisted three-phase partitioning of fermented BSG proteins yielded higher WHC (4.4–4.9 g/g) and OHC (7.3–8.3 g/g), demonstrating that microwave treatment enhances both water- and oil-binding properties [17].

#### 3.2.3. Emulsifying Properties

Proteins are widely used as emulsifiers in food systems because they can migrate to interfaces, orienting their polar and non-polar amino acid residues toward the water and lipid phases, respectively, forming a stable coating around droplets [79]. The emulsifying properties of proteins are typically evaluated through emulsion capacity (EC) and emulsion stability (ES). However, various parameters are also used to characterize these properties, such as the emulsion capacity index (ECI), emulsion activity index (EAI), emulsion stability index [24], and emulsion volume index [24,83].

**Figure 4:**
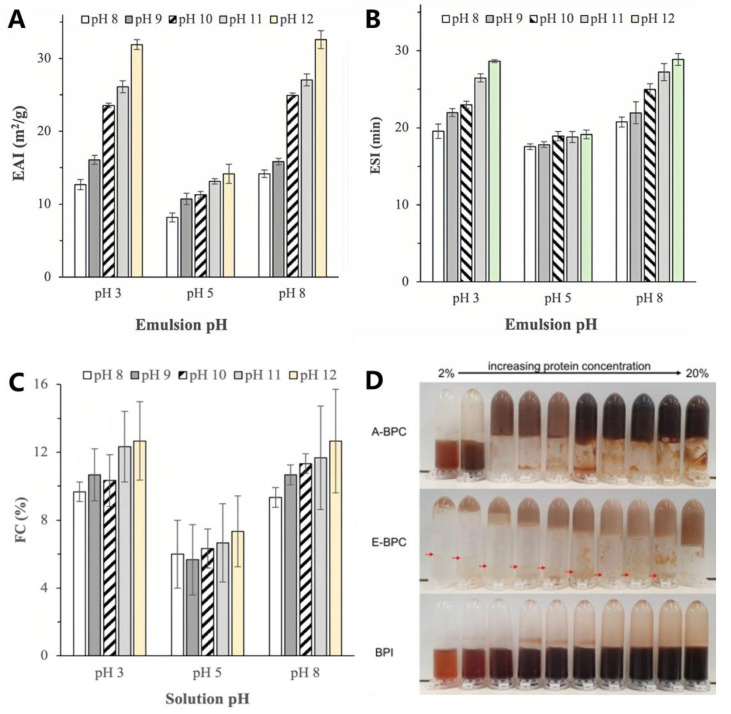
Emulsifying activity index (EAI) (**A**), emulsifying stability index (**B**), and foaming capacity (FC) (**C**) of BSG protein extracted at different pHs [73], Gelation of BSG proteins obtained by different extraction methods. The red arrows in E-BPC indicate the excess liquid at the bottom of the tube when inverted (**D**) [12].

Extraction PH and temperature are considered to be factors influencing emulsifying properties. Silva et al. [15] indicated that under conditions of pH 8 and 60 °C, alkaline extraction resulted in the best EAI (81.97 m^2^/g) and ESI (approximately 90 m^2^/g), suggesting that mild extraction conditions may better preserve the emulsifying properties of proteins. However, Hadinoto et al. [73] indicated that higher extraction pH would be helpful to obtain better emulsifying properties, as shown in Figure 4A,B.

Additionally, different extraction methods also have a significant impact on the emulsifying properties of proteins. Chin et al. [12] compared proteins extracted using alkaline extraction and ethanol extraction methods. The BSGP extracted by alkaline showed higher EAI, likely due to the exposure of polar amino acids and the presence of glutenin proteins that formed a stable protein network. In contrast, BSGP extracted by ethanol, which was more aggregated, exhibited lower emulsifying properties. Also, there were researchers who employed subcritical water extraction and obtained lower oil-water interfacial tension compared to alkaline and alcohol extractions, demonstrating stronger adsorption at the oil-water interface, which is crucial for forming stable emulsions [28]. Physical assistive methods such as ultrasound and microwave-assisted extraction are considered beneficial for enhancing emulsifying capacity. Li et al. [26] used ultrasound-assisted extraction to achieve higher EAI and ESI. As ultrasound power increased, EAI and ESI also increased, reaching a peak at 250 W. The reduction in protein size and the exposure of hydrophobic groups through ultrasound treatment enhanced protein adsorption at the oil-water interface, resulting in improved emulsifying properties [84,85]. Also, Chai and Chen [17] pointed out that microwave treatment during the extraction process is beneficial for achieving higher EAI and ESI.

Methods that promote smaller protein fractions and hydrophobic group exposure generally lead to improved emulsifying activity and emulsion stability. Chin et al. [31] addicted that solid-state fermentation followed by extraction significantly improved the emulsifying properties of BSG proteins. The fermentation process produced peptides with better emulsifying ability and increased hydrophobic interactions in the protein, helping it form more stable emulsions. Similarly, researchers [9] used enzymatic hydrolysis on BSGP, which also improved its emulsifying properties. One factor that affects this improvement is the type of enzyme used. Hydrolysates prepared with Alcalase and Pepsin showed a decrease in emulsifying activity (EAI) as the DH increased, indicating that excessive hydrolysis may reduce emulsifying efficiency. In contrast, Flavourzyme-hydrolyzed proteins maintained good emulsifying properties regardless of the DH, likely due to the enzyme’s ability to generate larger peptides with high surface hydrophobicity that adsorb well at the oil-water interface.

#### 3.2.4. Foaming Properties

Foam is defined as a two-phase mixture in which the gas phase is surrounded by the continuous phase (liquid or solid) [79]. Proteins are known for their ability to form and stabilize foam [86], and BSGP has thus been studied as a potential foaming agent. Foaming ability is generally assessed by foaming capacity (FC) and foaming stability (FS).

Studies have shown that the extraction method can significantly affect the foam-forming ability of proteins. The extraction PH is an important factor. As shown in Figure 4C, higher extraction pH can result in better foaming properties [73], possibly due to the higher surface hydrophobicity. Physical methods like ultrasound-assisted extraction (UAE) and microwave-assisted extraction (MAE) contribute to enhancing foam stability and foaming capacity. For example, UAE exposes more hydrophobic groups and reduces protein size, which allows proteins to adsorb better at the gas–liquid interface and improves foaming properties [26]. Similarly, MAE improves foaming properties by partially unfolding proteins and increasing their flexibility, which leads to stronger foam formation [17].

Enzymatic hydrolysis also improves foaming. Enzymes like Flavourzyme break proteins into smaller peptides, which can stabilize foam better because of their small size and higher hydrophobicity. Proteins treated with Flavourzyme show better foam stability than those treated with Alcalase or Pepsin because they form stronger foam films [9]. The degree of hydrolysis (DH) is important, with moderate DH levels leading to the optimal balance between peptide size and hydrophobicity, which improves both foaming capacity and foam stability.

#### 3.2.5. Gelation Properties

Protein gels are three-dimensional cross-linked networks of protein molecules. These gels can incorporate water, fats, sugars, and other components [79,87]. Gelation happens when proteins partially denature, which changes their shape and exposes active sites. This allows proteins to interact, aggregate, and form a gel [88]. Research has been conducted on the gelation properties of BSGP.

Silva et al. [15] showed that BSGP concentrates extracted at higher pH and temperature had better gelation. The partial protein denaturation exposed hydrophobic regions, which helped proteins interact and form a stronger, elastic network [80].

In addition, Hellebois et al. [77] found that a 5% protein concentrate from alkaline-extracted BSGP could form a viscoelastic gel when treated with glucono delta-lactone to lower the pH to 4.2. Heat treatment also improves gelation, especially under acidic conditions. Proteins subjected to heat were observed to form stronger gels due to protein aggregation and the formation of cross-linked networks, which is essential for gel strength and stability [89]. These findings suggest that heat treatment can facilitate gel formation by promoting the aggregation of proteins into structured networks.

Also, Chin et al. [12] highlighted the impact of different extraction methods, comparing alkaline extraction with ethanol extraction. Their results showed that alkaline-extracted proteins exhibited superior gelation properties, which is presented in Figure 4D. This could be a result of the alkaline extraction, in which hemicellulose (arabinoxylan) could be co-extracted and contribute to improved gelation as these biopolymers retain water very easily [90]. Furthermore, the presence of phenolics increased the solubility and WHC of the proteins, which in turn influenced their gelation, as previously demonstrated in sunflower protein isolate [91].

## 4. Conclusions and Future Perspectives

BSGP, as a sustainable and high-value plant-based protein source, holds broad application potential in future food systems. This review summarizes the latest advancements in various extraction methods, including conventional and novel approaches. These methods have shown excellent performance in enhancing extraction yield. However, the complexity of the BSG structure and the difficulty in separating the protein from other components, which leads to moderate purity, remain challenges in the extraction process. Furthermore, the variations in extraction yield and protein purity observed across different methods indicate that processing conditions still need further optimization for large-scale industrial production.

This review also highlights how various extraction parameters influence the structure and techno-functional properties of BSGP, emphasizing that extraction is not only about recovering protein but also about determining its functional characteristics. However, research exploring the connections between extraction techniques, protein structure, and the functional properties of BSGP remains limited. Similar to many other plant-based proteins, BSGP exhibits only moderate functional performance, which continues to restrict its broader application within the food industry.

It is worth noting that this review focused on extraction efficiency, purity, and techno-functional properties, without assessing nutritional aspects. Although literature indicates that alterations in protein structure properties may influence the nutritional quality of proteins, this relationship was not evaluated here due to the scope of the review and the limited number of studies addressing this aspect.

Therefore, future studies should place greater emphasis on elucidating the mechanistic pathways through which extraction methods influence protein functionality, and on employing protein modification strategies—such as enzymatic, physical, or chemical approaches—to enhance the techno-functional properties of BSG proteins for improved applicability in food systems. Also, the nutritional qualities of BSGP should be explored.

## Figures and Tables

**Table 1:** Composition of brewer’s spent grain.

Component (% Dry Basis)	[9]	[10]	[11]	[12]	[13]	[14]	[15]
Protein	26.7	23.10	22.8	22.7	22.5	22.44	17.87
Total fiber	n.d.	34.0	n.d.	64.3	45.8	n.d.	n.d.
Cellulose	n.d.	n.d.	17.1	n.d.	n.d.	20.56	12.31
Hemicellulose	22.5	n.d.	13.1	n.d.	n.d.	25.97	26.28
Lignin	n.d.	23.39	19.4	n.d.	n.d.	19.57	3.48
Lipids	n.d.	13.51	11.0	9.0	7.2	5.30	6.72
Ash	3.3	3.29	4.7	3.9	4.4	3.54	2.33
Starch	1.0	1.48	n.d.	n.d.	n.d.	2.23	n.d.
Phenolics	n.d.	1.70	n.d.	0.09	n.d.	n.d.	n.d.

All values expressed in g per 100 g dry material (% *w*/*w*); n.d., not determined.

**Table 2:** Extraction techniques of BSGP.

Type of Technique	Extraction Technique	Pretreatment	Extraction Parameters	Extraction Yield (%)	Protein Purity (%)	References
Conventional Techniques	Hydrothermal extraction	-	Mixed with water (1:10 *w*/*w*), stirred at 40 °C, 2 h, centrifuged, supernatant collected	6.8	40.7	[28]
	Alkaline extraction	Dried, ground at 22,000 rpm	Mixed with 110 mM NaOH (1:20 *m*/*v*), stirred, 50 °C/20 °C 1 h; Ph→3.8, centrifuged to collect precipitated protein	Pale BSG: 59.0 (50 °C) 28.55 (20 °C) Black BSG: 15.26 (50 °C) 11.0420 °C)	Pale BSG: 46 (50 °C) 45.74 (20 °C) Black BSG: 17.22 (50 °C) 19.42 (20 °C)	[10]
		Autoclaved (121 °C, 15 min), dried, ground (125–250 μm)	Mixed with 0.01 M NaOH (1:15 *w*/*v*, pH 12.4), extracted (60 °C, 30 min), centrifuged, repeated 3×, freeze-dried	45	27	[29]
		Autoclaved. Dried, ground, defatted with methanol-chloroform (1:2 *v*/*v*, 15:1 *w*/*v*), stirred (1 h), filtered, dried (60 °C)		38	Approximately 27	
		Autoclaved. Dried, ground, delignin with 60% ethanol (1:4 *w*/*v*), heated (180 °C under reflux, 90 min), filtered, washed, and dried		Approximately 45	32	
		Dried at 60 °C for 6.5 h to ~5.9% moisture	3 g BSG placed in a 33 mL extraction tank, 0.05 M NaOH. 40 °C, 60 min 103.4 bar	Approximately 19	69.7	[25]
			3 g BSG placed in a 33 mL extraction tank, 0.1 M NaOH. 40 °C, 60 min 103.4 bar	Approximately 22	Approximately 65	
		Dried,0.5% H_2_SO_4_,100 °C 20 min; pH adjusted to 3, left overnight; centrifuged to separate supernatant; dried	3 g BSG placed in a 33 mL extraction tank, using 0.1 M NaOH as the extraction solvent. Extracted at 40 °C, 60 °C, and 80 °C for 60 min under a pressure of 103.4 bar	40 °C: 37.6 60 °C: 57.5 80 °C: 65.3	40 °C: 60 60 °C: 50 80 °C: 32	
		Dried Mixed with water (1:20 *w*/*v*), ultrasound (frequency 37 kHz, power 100%,20min,30 °C)		40 °C: 24.1 60 °C: 40.9 80 °C: 53.8	40 °C: 66 60 °C: 46 80 °C: 25.2	
		-	Adjusted pH to 12, reacted for 60 min, centrifuged to collect the supernatant, and freeze-dried to obtain protein	66.41	-	[30]
		Stirred for 1 min; Mixed with distilled water (1:4, *w*/*v*), heated to 50 °C, pH 4.5, added cellulase (1:50), incubated for 60 min, inactivated at 85 °C for 20 min, centrifuged, and collected the precipitate		50.18	-	
		Dried and milled to a particle size of 10–200 μm	pH11, 60 °C, SSR (solid/solvent ratio) 1:17, 3 h	87	44.37	[15]
		-	Mixed with 0.1 M NaOH, pH > 11, SSR 1:10 (*w*/*w*), 40 °C, 2 h, centrifuged, and collected the supernatant	21.4	60.2	[28]
			Protein from the liquid phase			
	Ethanol extraction	-	Extracted with ethanol-sodium sulfite (pH 9) at 60 °C for 2 h. Centrifuged, precipitated protein with HCl, removed ethanol by rotary evaporation, centrifuged, washed, and freeze-dried	-	60.7	[31]
		BSG fermented with Rhizopus oligosporus (7 log_10_ cfu/g, 10:1 *w*/*v*) at 37 °C for 72 h. Dried and milled			66.2	
	Enzymatic extraction	Air-dried, milled, and sieved to 300 μm, treated with Depol 740 L (xylanase activity) at 50 °C for 5 h. Centrifuged, collected the precipitate	Mixed the precipitate with pH 9.5 sodium carbonate. Incubated at 50 °C for 4 h, centrifuged, and extracted protein from the liquid phase	53	-	[11]
			Mixed the precipitate with water (1:10, *m*/*v*). Incubated with Alcalase 2.4 L, Promod 144 GL, and Acid Protease A at pH 9.5, 6.5, and 3.5 (40 °C, 4 h), centrifuged, and extracted	Alcalase 2.4 L: 86 Promod 144 GL: 31 Acid Protease A: 40	-	
		Mixed with water (1:10, *m*/*v*), sheared at 24,000 rpm for 10 min, incubated with β-glucosidase (75 μg/BSG) at 50 °C, pH 5.0, for 4 h, inactivated at 80 °C for 20 min, and centrifuged (2700× *g*, 10 °C, 10 min)	The solid was treated with Alcalase 2.4 L (1:50) and Flavourzyme 500 L (1:100) at 50 °C for 4 h, inactivated at 80 °C for 20 min, and centrifuged. Remaining solid was mixed with water (7:100, *m*/*v*), stirred at 50 °C for 30 min, centrifuged, and freeze-dried	63	44	[32]
		Dissolved at 4 °C and milled	Mixed with deionized water to prepare a 5% (*w*/*w*) slurry. Added Alcalase protease (20 μg/g dry BSG), incubated at 60 °C for 4 h. Then vibrated for 15 min. Collected the filtrate, dried at 60 °C for 24 h, and stored at −20 °C	43.7%	42.8%	[33]
Novel Techniques	Ultrasound-assisted extraction		Mixed with 110 mM NaOH (1:20, *w*/*v*), ultrasound parameters: 70% amplitude, 15 min × 2.60 °C. Centrifuged to collect the supernatant, adjusted pH to 3.8, centrifuged to collect the precipitate, resuspended the particles, and freeze-dried	43%	-	[34]
		Dried and milled	Extracted at room temperature for 81.4 min with ultrasonic power of 88.2 W/100 mL, using 2.0 g BSG/100 mL pH 10 sodium carbonate solution	96.4 mg/g (dry BSG) Yield Approximately 45	-	[35]
		Dried at 50 °C, milled, and filtered through a 335 μm sieve	Mixed with 110 mM NaOH (1:15, *w*/*v*). Ultrasound-treated (20–25 kHz, 250 W, 25 °C, 20 min, 60% duty). Centrifuged, adjusted to pH 3.8, centrifuged, dissolved in 2 M NaOH (pH 7), dialyzed (1000 Da, 4 °C, overnight), and freeze-dried	86.16%	57.84%	[26]
		-	Mixed BSG with pH 10 sodium carbonate buffer, ultrasound-treated for 1 h, filtered, and centrifuged (10,000× *g*, 4 °C). Concentrated using 5 and 30 kDa membranes (25 psi, 25 °C), then freeze-dried	30 kDa: 10.01% 5 kDa: 14.09%	30 kDa: 15.98% 5 kDa: 20.09%	[36]
	Microwave-assisted extraction	BSG dried at 60 °C to <3% moisture and milled to <1 mm particle size	Mixed with 0.5 M NaOH solution (1:10, *w*/*v*), microwaved to 110 °C, and extracted for 10 min. Centrifuged to separate and collect the supernatant	93.99%	-	[37]
		BSG fermented with Rhizopus oligosporus (7 log_10_ cfu/g, 10:1 *w*/*v*) at 37 °C for 72 h. Dried and milled	MATPP process: Mixed BSG with water, stirred, and microwaved in a covered beaker. Filtered through fine cloth to obtain crude extract. Added saturated ammonium sulfate and t-butanol to the extract, vortexed for 3 min, left at room temperature for 30 min, and centrifuged (1000× *g*, 15 min). Separated the organic upper layer and aqueous middle phase, then freeze-dried	82.2%	-	[17]
	Subcritical Water Extraction	Washed, dried at 45 °C for 3 h	12 g BSG in a fixed-bed reactor (20.6 cm length, 2.8 cm diameter) at 5 MPa; water flow rate 4 mL/min; extracted at 185 °C for 150 min	78%	-	[21]
		0.5 mm Washed, dried at 45 °C to 8% moisture; ground to <0.5 mm particle size	Laboratory scale: 0.5 L reactor, BSG-water ratio 1:20 (*w*/*v*), 170 °C, stirred at 500 rpm, 5 MPa for 22 min. Pilot scale: 20 L reactor, BSG-water ratio 1:20 (*w*/*v*), 170 °C, 2 MPa for 22 min	Laboratory scale: 63% Pilot scale: 64%	Laboratory scale: 6.5 g/L Pilot scale: Not reported	[38]
	Pressurized Solvent Extraction		1.5 g BSG,9 g sand→10 mL extraction cell, preheat for 6 min at 1500 psi, 4.7% ethanol, 155 °C, 10 min, 5 cycles	69%	-	[34]
	Deep eutectic solvent Extraction	Defatted with supercritical CO_2_	Extracted using a solvent of 90 wt.% sodium acetate (NaAcO):urea (molar ratio 1:2) with 10 wt.% water. Solid-to-solvent ratio was 1:9 (*w*/*w*). Stirred at 80 °C for 2 h, filtered, washed, dialyzed using a 3.5 kDa membrane, then concentrated and dried	79%	52–54.7%	[39]

**Table 3:** Techno-functional properties of BSGP.

Techno-Functional Properties	Extraction	Treatment	Key Findings	Reference
Solubility	Alkaline	Extraction at varying pH values and temperatures	higher extraction pH → surface hydrophobicity ↑, →intermolecular hydrophobic interactions → solubility ↓ increasing temperature → promoting molecular motion and interactions with the solvent → solubility ↓	[15]
	Enzymatic, fungal fermentation	Enzymatic hydrolysis, Rhizopus oligosporus ATCC 64,063 fermentation	Peptides with better solubility, proteins with more charged amino acids → solubility ↑	[13,31,58]
	Microwave-assisted	Microwave-assisted extraction with three-phase partitioning for fungal-fermented BSG	Microwave treatment did not change the molecular weight, and solubility slightly ↑	[17]
WHC, OHC	Alkaline	Extraction at varying pH values and temperatures	pH from 8 to 12 → WHC ↑ (3.2 g/g to 5 g/g), due to compositional changes in the protein extracts. Temperatures ranging from 40 °C to 80 °C did not significantly affect the WHC, no significant effect on OHC	[15]
	Alkaline and alcohol extraction	Extraction via alkaline and alcohol	Alkali-extracted BSGP → exposed polar amino acid side chains contain high-molecular-weight glutenins, which form a network capable of retaining more water → higher WHC no significant effect on OHC	[12]
	fungal fermentation	Rhizopus oligosporus ATCC 64,063 fermentation	solid-state fermentation → increased number of polar groups → better WHC, OHC	[31]
	Ultrasound-assisted	110 mM NaOH as solvent, ultrasound treatment	Ultrasound treatment → exposure of hydrophobic groups buried within the protein molecules, α-helix content↓, protein unfolding, allows for better adsorption at water-oil interfaces → better OHC(3.1 g/g)	[26]
	Microwave-assisted	Microwave-assisted extraction with three-phase partitioning for fungal-fermented BSG	Microwave treatment enhances both WHC (4.4–4.9 g/g) and OHC (7.3–8.3 g/g)	[17]
Emulsifying Properties	Alkaline	Extraction at varying pH values and temperatures	Mild conditions(pH 8 and 60 °C) resulted in the best EAI (81.97 m^2^/g) and ESI (approximately 90 m^2^/g)	[15]
	Alkaline and alcohol extraction	Extraction via alkaline and alcohol	Alkali-extracted BSGP → the exposure of polar amino acids and the presence of glutenin proteins that formed a stable protein network → better emulsifying properties	[12]
	Subcritical water, alkaline, and alcohol extraction	Extraction via subcritical water, alkaline, and alcohol	subcritical water extraction → lower oil-water interfacial tension → stronger adsorption at the oil-water interface	[28]
	Ultrasound-assisted	110 mM NaOH as solvent, ultrasound treatment	Ultrasound treatment → the reduction of protein size and the exposure of hydrophobic groups → protein adsorption at the oil-water interface ↑ → improved emulsifying properties	[26]
	Microwave-assisted	Microwave-assisted extraction with three-phase partitioning for fungal-fermented BSG	Microwave treatment enhances both EAI and ESI	[17]
	fungal fermentation	Rhizopus oligosporus ATCC 64,063 fermentation	solid-state fermentation → peptides with better hydrophobic interactions → better emulsifying properties	[31]
	Enzymatic treatment	Enzymatic hydrolysis(Alcalase and Pepsin/Flavourzyme)	Alcalase and Pepsin → EAI ↓ as the DH increased; Flavourzyme-hydrolyzed proteins→maintained good emulsifying properties regardless of the DH, likely due to the enzyme’s ability to generate larger peptides with high surface hydrophobicity that adsorb well at the oil-water interface	[9]
Foaming Properties	Alkaline and alcohol extraction	Extraction via alkaline and alcohol	Alkali-extracted BSGP → the exposure of hydrophobic amino acid side chains and the presence of larger glutenin proteins → stronger and more stable foams	[12]
	Enzymatic treatment	Enzymatic hydrolysis(Alcalase and Pepsin/Flavourzyme)	Enzymatic hydrolysis → broke proteins into smaller peptides, which have a smaller size and increased hydrophobicity → better foaming properties Flavourzyme-treated proteins showed better foam stability compared to those hydrolyzed by Alcalase or Pepsin	[9]
	Ultrasound-assisted	110 mM NaOH as solvent, ultrasound treatment	Ultrasound treatment → better protein adsorption at the gas–liquid interface → improved foaming properties	[26]
	Microwave-assisted	Microwave-assisted extraction with three-phase partitioning for fungal-fermented BSG	Microwave treatment enhances both FC and FS by inducing partial protein unfolding and increasing protein flexibility	[17]
Gelation Properties	Alkaline	Extraction at varying pH values and temperatures	Higher pH and temperature → partial protein denaturation and exposure of hydrophobic regions → promoted intermolecular interactions and the formation of a stronger elastic network → enhanced gelation	[15]
	-	Heat treatment	Heat treatment can facilitate gel formation by promoting the aggregation of proteins into structured networks	[77]
	Alkaline and alcohol extraction	Extraction via alkaline and alcohol	Alkaline extraction → hemicellulose being co-extracted → ability to retain water → better gelation properties	[12]

## Data Availability

The original contributions presented in the study are included in the article, further inquiries can be directed to the corresponding author.

## Abbreviations

The following abbreviations are used in this manuscript:

BSG	brewer’s spent grain
BSGP	brewer’s spent grain protein
UAE	ultrasound-assisted extraction
MAE	microwave-assisted extraction
DES	deep eutectic solvent
HBA	hydrogen bond acceptors
HCA	hydrogen bond donors
WHC	water-holding capacities
OHC	oil-holding capacities
EC	emulsion capacity
ES	emulsion stability
EAI	emulsion activity index
ESI	emulsion stability index
ECI	emulsion capacity index
EVI	emulsion volume index
FC	foaming capacity
FS	foaming stability
